# Factors in Oncogenesis: Viral Infections in Ovarian Cancer

**DOI:** 10.3390/cancers12030561

**Published:** 2020-02-29

**Authors:** Sudipta Pathak, Jacek R. Wilczyński, Edyta Paradowska

**Affiliations:** 1 Laboratory of Virology, Institute of Medical Biology of the Polish Academy of Sciences, 93-232 Lodz, Poland; spathak@cbm.pan.pl; 2 Department of Surgical and Oncological Gynecology, Medical University of Lodz, 90-419 Lodz, Poland; jrwil@post.pl

**Keywords:** ovarian cancer, human papilloma virus, cytomegalovirus, review

## Abstract

Ovarian cancer (OC) is one of the leading causes of cancer death in women, with high-grade serous ovarian cancer (HGSOC) being the most lethal gynecologic malignancy among women. This high fatality rate is the result of diagnosis of a high number of new cases when cancer implants have already spread. The poor prognosis is due to our inadequate understanding of the molecular mechanisms preceding ovarian malignancy. Knowledge about the site of origination has been improved recently by the discovery of tube intraepithelial cancer (TIC), but the potential risk factors are still obscure. Due to high tumoral heterogeneity in OC, the establishment of early stage biomarkers is still underway. Microbial infection may induce or result in chronic inflammatory infection and in the pathogenesis of cancers. Microbiome research has shed light on the relationships between the host and microbiota, as well as the direct roles of host pathogens in cancer development, progression, and drug efficacy. While controversial, the detection of viruses within ovarian malignancies and fallopian tube tissues suggests that these pathogens may play a role in the development of OC. Genomic and proteomic approaches have enhanced the methods for identifying candidates in early screening. This article summarizes the existing knowledge related to the molecular mechanisms that lead to tumorigenesis in the ovary, as well as the viruses detected in OC cases and how they may elevate this process.

## 1. Introduction

Ovarian cancer (OC), the eighth most prevalent women’s cancer across the world, is a plethora of malignancies in the ovaries, fallopian tubes, and peritoneal cavity, which has been divided into two distinct types on the basis of histology, molecular pattern, and clinical outcomes. As per the GLOBOCAN study, 295,414 cases, 184,799 deaths, and 762,663 cases with 5-year survival were associated with ovarian cancer in 2018 [1]. Asian countries have the highest morbidity (51.6%) and mortality (49.9%) rates, while European countries hold the second highest morbidity (23.1%) and mortality (24.2%) rates [1]. Such poor survival rates result mostly from the fact that two-thirds of new cases are diagnosed at the FIGO stage III/IV, when cancer implants are already beyond the pelvis. Poor prognosis of this cancer type also owes to our inadequate understanding of the molecular mechanisms preceding ovarian malignancy. Ovarian cancer shares commonality in symptoms with gastrointestinal, genitourinary, and gynecological conditions, which restrains us from spotting signs specific to OC [2]. Asymptomatic early stages and failure in the identification of precursor lesions delay tumor detection, which hinders diagnosis at advanced stages (i.e., during primary peritoneal or fallopian tube carcinoma) [3]. The emergence of drug resistance and cancer relapse have limited the efficacy of the associated treatments, thus demanding identification of the markers that contribute to such events [4]. Although the early stages—for at least some of OC— seem to be identified as tube intraepithelial cancer (TIC), precise knowledge about the initiation of TIC and its mechanisms of progression to invasive OC is still lacking. The majority of TICs are identified in the distal region of the fallopian tube (fimbria) that is exposed to the pelvic cavity. This suggests that carcinogenesis may be promoted or driven by several factors, including the tumor microenvironment (TME), pelvic inflammatory disorder (PID), hormonal and genetic changes. Potential risk factors can aid in identifying plausible targets for early phase detection. Advancements in genomic and proteomic approaches have improved methods for the identification of candidate biomarkers and helped to develop effective screening strategies for early detection [5]. Molecular profiling and individualized OC treatment have provided optimism regarding the role of individualized medicine for oncological patients [6].

Microbial infections are currently recognized as potent risk factors in some cancer types and are estimated to account for 15%–20% of all cancer cases [7]. According to the International Agency for Research on Cancer (IARC), six oncoviruses are involved in malignant cell transformation, including human papilloma virus (HPV), hepatitis B and C viruses (HBV and HCV), human T lymphotrophic virus type 1 (HTLV-1), Epstein–Barr virus (EBV), and Kaposi sarcoma herpesvirus (KSHV) [1]. Viruses can act as direct carcinogens, by infecting cells undergoing neoplastic transformation, or as indirect carcinogens, by causing chronic inflammation and immune suppression [8]. The human immunodeficiency virus type 1 (HIV-1) increases cancer risk indirectly, mostly through immune deficiency. Retroviruses carry oncogenes derived from cellular genes involved in mitogenic signaling and growth control, while DNA tumor viruses encode oncoproteins important for cell transformation [9]. Viruses have developed many strategies to evade recognition and the antiviral immune responses of the host. They may play the role of cofactors and potentiate the oncogenic effect of other pathogens. There is currently no evidence that viruses have the pathogenic capacity to give rise to ovarian cancer.

## 2. Ovarian Carcinogenesis 

Ovarian tumors comprise a heterogeneous group of lesions, arising either from epithelial, germ cell, or sex-cord stromal tumors. Histologically, about 90% of ovarian tumors have occurred through the transformation of epithelial cells and are thus designated as epithelial ovarian cancer (EOC) [10]. There are four histological subtypes of EOC: serous, mucinous, clear-cell, and endometrioid. Low-grade and high-grade serous ovarian carcinomas (LGSOC and HGSOC, respectively) of the ovary are two different neoplasms, with distinct sites of origin, modes of carcinogenesis, and molecular–genetic features [11,12]. Approximately 10% of ovarian malignancies arise from germ cells, metastatic tumors, and unclassified tumors [10]. According to a dualistic model of ovarian carcinogenesis, EOC subtypes have been classified into two main categories: Type 1 neoplasms develop along a step-wise progression, from pre-malignant or borderline lesions, and display frequent oncogenic alterations in cellular signaling pathways, such as small GTPases (RAS), mitogen-activated protein kinases (MAPK), and the phosphoinositide-3-kinase-protein kinase (PI3K)-AKT. Type 1 tumors include: LGSOC, seromucinous, endometrioid, clear cell carcinomas, mucinous carcinomas, and malignant Brenner tumors [13]. However, it has been proposed that cancers associated with endometriosis and mucinous OC be considered as a separate group [14]. Type 2 neoplasms are aggressive with poor prognosis and are characterized by mutations in tumor suppressor p53 protein (*TP53*) gene and genomic instability. It has been shown that p53 can induce DNA repair proteins during DNA damage, cell cycle arrest at the G1 phase, apoptosis, and senescence [13]. HGSOC is a prototypical type 2 neoplasm, which is characterized by high-grade nuclear atypia with the potential for multinucleation. It was thought that HGSOC originated from ovarian surface epithelial (OSE) cells [15]; however, evidence has suggested that HGSOC originates from the distal end of the fallopian tube, ovary, or peritoneum [16,17,18]. Fallopian tube epithelium expressing *TP53* mutation undergoes malignant transformation to serous tubal intraepithelial carcinoma (STIC) [19]. Over 90% of HGSOC tumors have an alteration in *TP53* gene [20]. A STIC may transform into a malignant tumor due to its location and metastasize to the ovary and pelvic peritoneum. 

### 2.1. Ovulation

The main risk factors for HGSOC are the lifetime number of ovulations: the ovary can be confronted with carcinogenesis if the number of lifetime ovulations correlates with an increased risk of OC [21]. Ovulation promotes the migration and adhesion of malignant cells to the ovary by disrupting the OSE, as well as releasing chemokines and cytokines [22]. The wound thus formed is repaired by the proliferation of the epithelial cells and such aggregation forms inclusion cysts, which have the potential to trigger ovarian carcinogenesis [15,18,23,24]. During ovulation, the malignant transformation of cortical inclusion cysts (CICs) in the ovarian cortex has been hypothesized to play a role in the progression of HGSOC. Recent studies have revealed that HGSOC arises from occult carcinomas in the fallopian tube, while a subset of HGSOC may develop from CICs derived from the implantation of the tubal epithelium when the OSE is disrupted at ovulation [25]. This is of clinical relevance, since HGSOC displays a morphology similar to serous carcinomas arising in the female genital tract encompassing the presence of papillary or glandular structures and cytologic atypia [26]. Epidemiological data have shown that estrogen—which, under normal conditions, regulates the function of OSE—has an influence on the progression of OC [27]. Syed et al. [28] showed that estrogen poses a paradoxical role in the expression of tumor suppressor genes and oncogenes. Within normal OSE, estrogen is known to promote cellular growth in a non-oncogenic direction and reparation of follicular ruptures. However, within malignant OSE, it begins to support the course of ovarian carcinogenesis by decreasing the expression of genes associated with the stable growth of cells and enhancing the expression of genes associated with oncogenic potential [28]. Regulatory hormones and growth factors which display dichotomous functions in the ovary have provided effective targets for hormone therapies and growth blockers in ovarian cancer treatment [29,30].

### 2.2. Inflammation

Inflammation is an important factor in promoting ovarian cancer cell proliferation and metastasis. Ovulation, PID, endometriosis, polycystic ovarian syndrome, and obesity are major sources of inflammation in ovarian and fallopian tube tissues [31,32]. In vivo stimulation of ovulatory wound repair, ovulation, and ovarian surface scarring increases the seeding of cancer cells and infiltration of immune cells at the wound site and tissues surrounding the ovary. Inflammatory events during ovulation and ovulatory wound repair contribute to an increased risk of OC [33]. 

Chronic inflammation in the peritoneum results in the activation of signaling pathways, and immune responses, thus emerging as an important risk factor for EOC. It potentiates the transformation of cells from normal to malignant in fallopian tube and ovary. Data have supported that the overexpression of inflammatory pathways promotes EOC tumorigenesis, and resistance to chemotherapy. Several factors, such as interleukins (e.g., IL-1, IL-6, IL-8), tumor necrosis factor type α (TNF-α), and others are produced by tumor cells and activated by immune cells in tumor milieu [34,35,36]. The activation of the innate immune response employs macrophages and dendritic cells (DCs) to secrete chemoattractants, including IL-8 and monocyte chemotactic protein-1 (MCP-1) and usher the recruitment of neutrophils, lymphocytes, and natural killer (NK) cells, which produce reactive oxygen species (ROS), cytokines, chemokines, and growth factors [32]. High oxidative stress results in oxidative damage, which increases the expression of molecular drivers of mutagenesis, such as activation-induced cytidine deaminase (AID). AID subsequently introduces DNA damage, genomic rearrangements, and global hypomethylation, which contribute to the malignant transformation of OC [37]. The interaction of cytokines with Toll-like receptors (TLRs) at the tumor cell surface initiates the activation of proinflammatory pathways, mediated by nuclear factor kappa-light-chain-enhancer of activated B cells (NF-κB), and the signal transducer and activator of transcription (STAT) [32]. The infiltration of tumor-associated macrophages (TAMs) into the TME is mediated by MCP-1 and colony-stimulating factor 1 (CSF-1). At the site of inflammation, the polarity of TAMs shifts to M1 phenotype through their interaction with interferon-γ (IFN-γ), TNF-α and TLR-mediated interaction with endotoxins (lipopolysaccharides, LPS), whereas M2 macrophages are stimulated by IL-4/IL-13, IL-10, and transforming growth factor-beta (TGF-β) [38,39]. M2 macrophages release immunosuppressive cytokines (IL10, TGF-β) which contribute to OC tumor progression [38]. High expression of programmed death ligand-1 (PD-L1) on M2 macrophages and the production of immune inhibitory molecules B7-H4 repress the adaptive immune response, inactivate the cytotoxic T-cell response, and are associated with advancement in OC tumor stage [40,41]. 

Pattern recognition receptors (PRRs), such as TLRs, primarily play a role in activating antigen-presenting cells (APCs) to stimulate cytotoxic responses, maintaining tissue repair at the ovulation site [42]. Conversely, tumorigenesis in OC is facilitated by the interaction of TLRs on tumor cells with different effector molecules: a) TLR5 with MyD88 [43]; and b) TLR9 with unmethylated CpG DNA motifs released from surrounding necrotic cells [44]. OSE expresses TLRs receptive to microbial pathogen-associated molecular patterns (PAMPs) in order to facilitate protection against common gynecological infections. However, the resilient infection exhibited by oncolytic pathogens holds the potential to downregulate the expression of and subdue the inhibitory pathways mediated by TLRs [45,46]. Zhou et al. revealed that the transcriptional profiles of genes involved in TLR and NOD-like receptors (NLR) signaling pathways were different between OC tissues and normal distal fallopian tubes [47]. 

In TME of HGSOC tumors, the collagen fibers allow CD8+ T-cells to cross the stromal barrier and engage with major histocompatibility complex (MHC) class I molecules on the epithelial cancer cells [48]. CD8+ T-cells cooperate with CD20+ B-cells to promote antitumor immunity, possibly by inhibiting the development of T-cell anergy [49]. The accumulation of regulatory T cells (Tregs) in TME, probably triggered by an inflammatory stimuli, may lead to significant immune suppression in the advanced stages of OC [50]. Release of IL-10 and TGF-β from Treg inhibits cytotoxic functions of T-cells via direct contact. Moreover, B7-H4+ overexpression on the OC cells and tumor-associated macrophages increase the ratio of Tregs/CD8+ T cells and associate with poor prognosis [51]. High Treg/T helper 17 (Th17) ratio promotes EOC progression and metastasis into the peritoneum [52]. Adoptive T-cell therapy for the conversion of Treg to proinflammatory Th17 and genetically modifying APCs with co-stimulatory ligand may enhance cytotoxic effects of T cells [53]. 

## 3. Microbiome in Ovarian Cancer 

The infectious nature of carcinogenic pathogens distinguishes them from other cancer-causing factors. In vitro models have demonstrated the carcinogenic potential of common bacterial (*Chlamydia trachomatis, Mycoplasma genitalium*) and viral (HPV) pathogens in the infection of female reproductive organs and PID [54]. There is currently no evidence to support the hypothesis that microorganisms have the capacity to give rise to OC. However, the increased risk of ovarian carcinogenesis in the fallopian tubes and PID may raise concerns about the involvement of pathogens in OC. Persistent infections induce chronic inflammation, angiogenesis, and immune response as a part of ovarian tumor pathogenesis. The ovaries and their neighboring parts in the female genitalia are highly susceptible to infection from outside pathogens via the peritoneal tract. In addition, chronic inflammation, a low count of APC, and anti-apoptotic capacity during the reparation of the epithelial surface cause the epithelial cells to be more susceptible to infection [55]. The upper reproductive tract is not sterile and has a unique microbiome which has received increased attention [47,56].

Profiling the microbiome composition has provided insights into the viruses in ovarian tumors [56]. It has shown signatures of *Retroviridae*, followed by *Hepadnaviridae*, *Papillomaviridae*, *Flaviviridae*, *Polyomaviridae* and *Herpesviridae* (human cytomegalovirus—CMV, EBV, KSHV, and HHV6) families [56]. Approximately 23% of these were identified as tumorigenic viruses and were prevalent in more than 50% of screened cancer samples. However, the heterogeneity of the microbiome in the ovary has posed a challenge; for example, in latent infections, as well as the differential pattern of microbial existence in ovarian tumors and surrounding ovarian tissues in the same patient [56]. It seems possible that immunosuppression in the local microenvironment of the tumor creates a milieu favorable to the persistence of specific viruses. During the two last decades, with the use of highly sensitive techniques, several groups have detected the presence of some viruses in OC cases. It should be noted that the detection of a viral genome in OC tissues is not sufficient to prove viruses as a causal factor in the genesis of disease. Viral sequences have been commonly detected in both non-malignant and malignant ovarian tissues. Moreover, there has been a lack of suitable animal model systems which mimic the progression of this human disease. Moreover, viruses detected in tumors from OC patients are strictly species-specific and cannot be propagated in vitro (i.e., HPV), which delays the development of in vivo models for viral infections. In Table 1, we present studies that have searched for viral infections in OC tissues, benign ovarian tumors, and tissues surrounding such tumors. 

HPV is one of the most common types of sexually transmitted virus in the world. In the United States, the prevalence of oral HPV among adults has been reported as 7.3%, while genital HPV prevalence was 42.5% [77]. The HR-HPV prevalence in China was 17.7% [78]. Tang et al. detected HPV in 96.6% of cervical, and 14.1% of head and neck, bladder urothelial, and lung squamous cell carcinomas [79]. Hitherto, the prevalence of HPV in the pathogenesis of OC is still controversial. Some reports have confirmed the presence of HPV in malignant ovarian cancer [56,58,61,62,63,64,65,66,67,68,70,71,72,73], while others did not [57,59,60] (Table 1). Differences in detection methods, low copy number of HPV DNA, sample types, small sample size, and geographic regions of sample origin were potential causes of such discrepancies. Studies from Asia have generally reported a higher prevalence of HPV in EOC tumor tissues than studies from Western countries [80,81]. Overall, the HPV prevalence in patients with OC was found as 17.5% [81]. High-risk HPV16 and HPV18 are predominant genotypes associated with advanced stages of OC [61,66,67,68]. Molecular signatures of high-risk HPV (HR-HPV) types, along with low-risk HPV (LR-HPV) types, mostly HPV6, were detected in OC samples, while LR genotypes were found in non-cancerous controls [71]. Banerjee et al. [56] found widespread integration of HPV sequences into the genome of tumor cells. Integration of HPV *E6* and *E7* oncogenes has been shown to trigger the degradation of tumor suppressing proteins (p53 and Rb) and induce tumorigenesis [82,83]. 

Hidden herpesviruses may play an important role in the ovarian environment. However, the correlation between herpesviruses and OC remains controversial. CMV is not a typical oncogenic virus, but it is known for its characteristic of oncomodulation to enhance tumor growth and migration [84,85]. Approximately 50% of ovarian cancers in the Indian population have been shown to be positive for CMV DNA, of which 80% were invasive and 20% were borderline tumors [65]. In contrast, no association was observed between CMV and OC in the Danish population [74]. It was suggested that tumor heterogeneity, the uneven distribution of infected cells, and differences in the methodological approaches might be a probable reason for no prevalence of CMV in ovarian tumor tissues despite involving a large sample-size study. Higher prevalence of EBV DNA was noted in patients with EOC, as compared to a benign control group [74]. The incongruent findings in the microbiome in OC could be due to the possibility that the involved pathogen was not present at the time point at which OC was diagnosed [86]. After the virus alters the gene expression pattern and heritable changes are triggered in the host cell, recurrent DNA recombinogenic activities occur that may lead to the loss of the viral genome from cells during neoplastic transformation. With respect to this speculation, methods that entail the study of “hit and run” oncogenic effects of the pathogen could be strategized for efficient detection [86]. 

Some studies have suggested the association of bacteria, including *Chlamydia*, *Mycoplasma* and *Brucella,* with OC [64,70,87]. Bacterial pathogens develop chronic, asymptomatic infections and enhance the risk of OSE neoplastic transformation. *Chlamydia trachomatis* is an obligate intracellular bacterium which causes an asymptomatic infection by preventing phagosome–lysosome fusion, evading the host’s immune response, degrading transcriptional factors, and impeding the expression of MHC and cytokines [88,89]. The mechanisms involved in bacterial infection, such as the inhibition of DNA damage repair proteins [84], degradation of p53 [90], and cell cycle disruption due to increased MAPK signaling [89], ensure the survival of the bacterium while fostering a predisposition to carcinogenesis [91]. *C. trachomatis* DNA was detected in 80% of OC samples [65]. *Mycoplasma genitalium* has been detected in pelvic inflammation and tubal factor infertility [92]. *Chlamydia* and *Mycoplasma* have also been found in at least 60% of screened OC samples [56,65,93]. *Neisseria gonorrhea* causes genital infections such as cervicitis/urethritis and pelvic inflammation and triggers DNA damage, downregulation in p53 expression, and arrest at G1 phase in the cell cycle [94]. However, a study carried out by Idahl et al. [95] showed no association of *M. genitalium* or *N. gonorrhea* with OC. 

## 4. The Role of Human Papillomavirus in Oncogenesis

HPV is a small, non-enveloped, DNA virus with a single double-stranded genome. Over 200 types of HPV have been identified. LR-HPV types, including HPV6 and HPV11, cause the formation of benign lesions (i.e., genital warts), but rarely give rise to cervical cancers. High oncogenic potential HPVs include mainly HPV16, 18, 31 and 45, and these types cause intraepithelial neoplasia (or flat warts), which advance to invasive cancers [96,97]. Among them, HPV16 most likely causes cervical, vaginal, vulvar, penile, and anal cancers, as well as cancers of the head and neck [97]. The role of HPV in the predisposition of cancer has not been restricted to these cancers but also in colon, lung, and breast cancers. Cancers with HPV-positive infection exhibit a distinctive etiology, carcinogenesis, and tumor biology (i.e., lower DNA content, smaller cell nuclei, basaloid growth of lesions), providing further challenges for detection [98]. In most cases, HPV infection resolves spontaneously, while in rare cases, the infection persists for several years. Productive infection generates as much as 100 copies of the viral genome in every cell, where viral genomes are maintained as episomes and amplified in daughter cells. If the infection is caused by an HR-HPV type, the infection can persist, leading to genomic instability and neoplastic transformation of the epithelium. 

The early region of HPV genomes encodes six proteins (E1, E2, E4–E7) necessary for viral replication, while the late region encodes two structural proteins (L1 and L2) which are required for virion assembly and transmission [96,99,100]. E6 and E7 act as oncogenes that promote tumor growth and malignant transformation, targeting several negative regulators of the cell cycle, including p53 and retinoblastoma protein (pRB), respectively. HPV takes the opportunity at the division of the epithelium, anchoring to surface molecules (heparin sulphate proteoglycans) on the basal epithelium to pave its way inside the cell by clathrin-mediated endocytosis; finally, the viral DNA inhabits the host cell nucleus in its episomal form [99,101]. Breakdown of the nuclear envelope and binding of L2 protein with dynein are required for the transport of HPV DNA to the nucleus [102,103]. E1 and E2 are expressed to maintain low expression levels of the E6 and E7 oncoproteins to avoid activation of tumor progression [99,101]. This thus raises the possibility for effective early-stage detection of HPV by targeting early proteins such as E1 and E2 when oncoproteins like E6 and E7 are expressed at low levels [104]. The HR-HPV E6 oncoprotein promotes proteasomal degradation of p53, eliminates the trophic sentinel response for viral DNA synthesis and upregulates telomerase activity to evade cell senescence [105,106,107]. E7 inactivates pRB to override p21-mediated growth arrest and upregulates p16 for the immortalization of cells [105,107]. E6 eliminates the “trophic sentinel” response, a defense mechanism against the replication of viral DNA induced in absence of a mitogen that helps in the synthesis of viral DNA and evasion of cell senescence [100]. The upregulation of telomerase activity by E6 in concert with the upregulation of p16 activity by E7 leads to escape from cell-death and the immortalization of epithelial cells [106]. E6 and E7 are required for maintaining malignant transformation while other viral proteins (e.g., E5) contribute to neoplastic proliferation in the early stages of viral infection. The persistence of HPV infection may last several years, which puts the host in danger of tumor progression [108].

**Figure 1:**
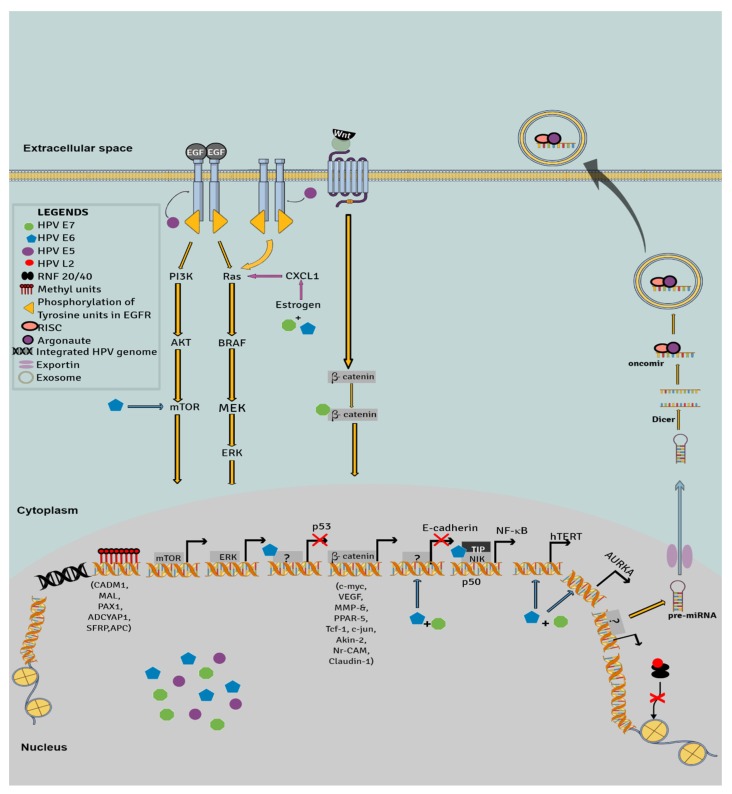
Signaling pathways deregulated in the presence of high-risk HPV. High-risk HPV infection increases cancer risk by: a) Increasing cell proliferation (PI3K/AKT mTOR, Ras-ERK, β-catenin pathways); b) Increasing cell proliferation and progression by the inactivation of p53 expression, E-cadherin and hypermethylation of tumor suppressor genes; c) Increasing immortalization of cancer cells by increasing telomerase activity and AURKA at G2/M phase; d) Increasing intercellular communication and epithelial-mesenchymal transition by upregulating the expression of oncomirs and inhibiting the interaction of histone proteins with ubiquitin ligases. **Abbreviations:** phosphoinositide 3-kinases (**PI3K**), serine/threonine-protein kinase (**AKT**), mammalian target of rapamycin (**mTOR**), epidermal growth factor (**EGF**), GTPase Ras (**Ras**), serine/threonine-protein kinase B-raf (**BRAF**), mitogen-activated protein kinases (**MEK** and **ERK**), chemokine (C-X-C motif) ligand 1 (**CXCL1**), telomerase reverse transcriptase (**hTERT**), aurora kinase A (**AURKA**), cell adhesion molecule 1 (**CADM1**), myelin and lymphocyte protein (**MAL**), paired box 1 (**PAX1**), adenylate cyclase activating polypeptide 1 (**ADCYAP1**), secreted frizzled-related protein (**SFRP**) and adenomatous polyposis coli (**APC**), NF-κB-inducing kinase (**NIK**), and TRAF-interacting protein (**TIP**).

Upon integration of HPV DNA into the host genome, E5 complexes with epidermal growth factor receptor (EGFR) to induce EGF-dependent activation of the mTOR pathway and EGF-independent activation of signaling cascades of MAP kinase p38 (regulating the expression of E6 and E7 genes in HPV16 infected cells) [109] and Erk1/2 pathway (for proliferation) [109,110,111,112] (Figure 1). E5 stimulates overexpression of c-jun, junB, and c-fos through AP-1 and NF-1, thus transactivating the viral genes *E6* and *E7*. The cellular transformation is further augmented by suppression of p21, complementing suppression of pRb by E7. E5 expression is coupled with the dephosphorylation of Connexin 43, as well as E5-stimulated c-jun and junB expression, impairing cell–cell contact and anchorage-dependent growth, one of the early characteristics of cancer cells [110]. The unique characteristics of viral transcription are the initiation of transcription at more than one promoter site and polycistronic pre-mRNA, causing the expression of multiple RNAs with many open reading frames. In addition to this event, the splicing mechanism maintains the proteomic diversity and generates isoforms of viral proteins E2 [113] and E6 [114]. The isoform of E6, E6*I, is considered to have anti-tumorigenic effects—it abrogates E6-mediated p53 degradation and help in initiating apoptosis [115]. E6*I has an antagonizing effect on E6 function and may alleviate the virulence of HPV in the infected cells. However, other effects of E6*I, such as the promotion of DNA damage and stabilization of E6 and E7, are involved in cancer development [116,117].

A meta-analysis on the prevalence of HPV in OC showed 15.5% (0%–67%) HPV-positive OC cases worldwide [80]. The presence of HR-HPV genotypes in not just the lower, but also upper genital tract can suggest that HPV may be involved in the development of EOC [68,71]. HR-HPV E6 and E7 have been found to be expressed in ovarian tumors [118]. Infection with HR-HPV renders low expression of p53 and high expression of p16 in OC; furthermore, the expression of E6 has been shown to be suggestive of low expression of p53 [119]. The integration of the HPV genome into the host genome leads to cell cycle disruption and increased genetic instability in the host (Figure 1). Arfi et al. found the same locus and the same nucleotide breaks for HPV18 DNA integration with high expression of p16, which is indicative of ovarian metastasis from cervical adenocarcinoma [120]. Examining the locus of viral–host integration will be beneficial to study the role of HPV in ovarian tumors for patients with a history of HPV-positive cervical cancer. 

In cervical cancer, HPV DNA integration in the host genome, genomic rearrangement, involving structural changes at the HPV DNA integration site, and the activation of replication at viral integrated sequences contribute to oncogenesis [121,122,123]. Genetic alterations which foster carcinogenesis involve DNA methylation and nucleotide polymorphisms. HPV-mediated promoter hypermethylation of tumor-suppressor genes, such as cell adhesion molecule 1 (CADM1), myelin and lymphocyte protein (MAL), paired box 1 (PAX1), adenylate cyclase activating polypeptide 1 (ADCYAP1), secreted frizzled-related protein (SFRP) and adenomatous polyposis coli, may have implications in the development of OC [71,121]. 

The activation of the PI3K/AKT/mTOR signaling pathway may play a key role in the virus–host interaction in HPV-positive cancer cells. HPV not only integrates within the host genome, but strongly associates with the host’s biochemical cascades to steer carcinogenesis. HPV proteins drive carcinogenesis quite contrarily—while E7 enhances the Wnt/β-catenin signaling pathway and yes-associated protein 1 (YAP1) expression, E2 suppresses E7 expression and makes β-catenin and its coactivators less available. It was shown that E1 induced the transcription of antioxidant response elements (ARE), increased the expression of cytoprotective genes, and pushed cancer cells towards chemoresistance. The invasiveness of cancer cells and epithelial-mesenchymal-transition (EMT) plasticity are promoted by the binding of L2 with RNF20/40, a ubiquitin ligase complex and a strong tumor suppressor [122]. HPV infection also triggers inflammatory pathways, primarily targeting the central regulators of inflammation, the NF-κB, STAT3, and cyclooxygenase-prostaglandin (COX-PG) pathways. In oral cancer, HPV E6 activates the NF-κB pathway by interaction with p50, NF-kappa-B-inducing kinase (NIK), and TRAF-interacting protein; in parallel, E7 inhibits apoptosis by targeting TNF-α [123]. In cervical cancer, the HPV E6-mediated elevated activity of IL-17 and increased expression of IL-1β by HPV E7 activate NF-κB [124]. Moreover, the stimulation of NF-κB by E6 induces the production of IL-6, which is required for the activation of STAT3 [125]. Although persistent HPV infection elevates inflammatory cytokines, IL-6, IL-8, TNF-α and MIP-1α, cervical cancer patients have been shown to display a decrease in lymphoproliferation against HPV infection [126]. The upregulated expression of COX-2 and PG-E by HPV E5, E6, and E7 provoke tissue remodelling, cellular proliferation, angiogenesis, and inhibition of apoptosis. 

Malignancy and the spreading of tumors can be supported by miRNAs, functioning as cargoes. The transport of miRNA, channeled through the extracellular matrix and body fluid, involves miRNA encapsulation in exosomes, their transporters and mediators of intercellular communication [127,128,129]. In HPV-infected cells, the continuous expression of E6/E7 oncoproteins boosted intracellular levels of oncomirs and increased their abundance in exosomes. As body fluids are easier to procure than tissues, profile of exosomal miRNAs can be studied as a basis for diagnosis [130]. 

HPV also seeks aid from glandular molecules, such as estrogen, that manifest as cocarcinogens. The expression of E6 and E7, elevated levels of estrogen and expression of estrogen receptor alpha (ERα) [131] stimulate the onset and progression of neoplastic lesions in transgenic mouse models with cervical cancer [132]. Working in synergy, HPV and estrogen increase the expression of proinflammatory chemokines such as CXCL1 [133]. Estrogen has also been shown to upregulate stromal cell-derived growth factor (SDF-1/CXCL12) [133]. Chemokines, such as CXCL1, are present at elevated levels in ascites in OC, promoting the upregulated expression of EGFR and extracellular signal-regulated kinases MAPK/ERK signaling [134]. However, the expression studies of CXCL1 and estrogen in HPV-positive OC are required to define a relation between estrogen and HPV.

In vitro studies based on the immortalization of OSE cells and OC cell lines with HPV-16 E6/E7 may throw light on OC development. Immortalization has been a crucial step in cell transformation and cancer progression [135]. The transfection of OSE cells with HPV-16 E6/E7 has been shown to introduce genomic instability, telomere dysfunction, resistance to anti-tumor agents and the development of malignant characteristics over time [136,137,138,139,140]. The development of isogenic cell line with HR-HPV E6 showed downregulation of p53, AKT/ERK-independent activation of mTOR signaling pathway, utilization of glutamine metabolism for cellular homeostasis and upregulation of calcium-dependent phospholipase A2, which could be involved with migration of cells [141].

## 5. Herpesvirus Infection in Ovarian Cancer

**Figure 2:**
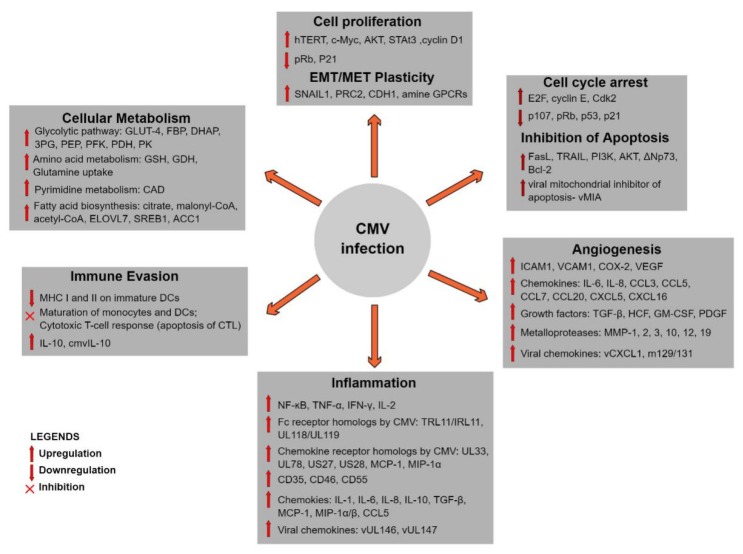
Signaling pathways deregulated during cytomegalovirus (CMV) infection. CMV may function as an oncomodulator to drive carcinogenesis. It seems to regulate the expression of proteins participating in cell proliferation, cell cycle arrest, cellular metabolism, apoptosis, angiogenesis, inflammation, immune suppression, and EMT plasticity [153,154,155,156,157,158,159,160,161]. It also codes for proteins that function as homologs for human proteins (such as chemokine receptors, Fc receptors, inhibitor of apoptosis).

Herpesviruses are large DNA viruses with a linear double-stranded DNA genome within an icosahedral capsid. These common pathogens can establish life-long infections and contain oncoproteins, promoting malignant transformation and metastasis. They affect cancer-implicated mechanisms, including tumor-promoting inflammation, immune evasion, and immunosuppression (Figure 2). Very few studies have examined the presence of herpesviruses DNA and proteins in OC tissues and, so far, evidence of CMV and/or EBV infection has been detected [65,71,72,73]. EBV has been recognized as oncogenic, while CMV has been implicated as an oncomodulator in malignant diseases from different cancer entities. The oncomodulatory properties of CMV may play an important role in ovarian carcinogenesis and disease progression. However, the role of herpesviruses in the development of OC is unknown.

CMV is a widespread opportunistic DNA virus with overall seroprevalence ranging from 40% to 100%, depending on age, socioeconomic status, and geographical location [142]. It has developed mechanisms that allow its survival in latent form in an immunocompetent host, reactivating during immunosuppression. CMV can spread to the upper genital tract and infection is usually persistent, latent, and asymptomatic. Several groups have detected CMV infection in various tumor malignancies, including colorectal cancer [143,144], malignant glioma [145], prostatic neoplasia and carcinoma [146,147], cervix cancer [148], breast cancer [149], and EBV-negative Hodgkin’s lymphoma [150]. CMV DNA and proteins have been commonly detected in OC, including HGSOC, and in benign cystadenomas [65,71,72,73]. The expression of CMV immediate-early (IE) and late tegument (pp65) proteins in most of the tissue sections from OC cases was found [72,73]. Moreover, these patients also had higher levels of CMV-IgG and IgM than healthy controls. The results show the presence of low amounts of CMV DNA in 50%–75% of tumor tissue samples obtained from women with EOC, while low protein expression or expression at different levels was found in most tissue sections, including those from patients with serous EOC [65,71,72,73]. In contrast, another study did not support any association between CMV and EOC [74]. It has been recently found that almost two-thirds of EOC patients demonstrated coinfection with both CMV and HPV16 in the pathological samples [71]. 

CMV is not considered to be oncogenic but, instead, acts an oncomodulator that may infect cancer cells and change the TME [151,152]. The onset of carcinogenesis begins with the CMV proteins IE1, IE2, pp71, and pUL97 phosphorylating pRB, inhibiting the cell cycle arrest functions of p53 and interfering with the cyclins, Wnt, PI3K/Akt, NF-κB, and STAT3 [153,154,155]. CMV proteins inhibit apoptosis and cell cycle arrest and promotes cell proliferation, angiogenesis, inflammation, and immune evasion [156,157,158,159,160,161]. CMV induces the expression of COX-2, NF-κB, and STAT-3, and the production of proinflammatory cytokines, prostaglandins, and leukotriens [156]. Moreover, translocated NF-κB interacts with the viral immediate early proteins (IE1-72, IE2-55, and IE2-86), transactivates promoters of NF-κB subunits, p105/p50, and p65, thereby elevating its activity [157,158]. For persistent infection, CMV evades immune recognition, downregulates MHC class I and II markers, and releases cmvIL-10, which impairs maturation of DC and stimulation of cytotoxic T-cells [153,157]. CMV infection in the endothelial cells of blood vessels induces the expression of the adhesion molecules ICAM and VCAM, which enhances monocyte trafficking and secretion of VEGF, a potent angiogenic factor. The interaction of CMV US28 with NF-κB elevates COX2, VEGF, and the migration of macrophages for promoting angiogenesis [158]. CMV strains have been shown to be capable of initiating oncogenesis in human breast epithelial cells by dysregulating p53, pRB, and telomerase [160]. CMV can activate epithelial cells, promote EMT and reverse the process (i.e., mesenchymal to epithelial transition) in tumor cells [160,161]. These CMV-promoted processes may potentially enhance metastatic tumor dissemination through the blood and lymphatic vessels and tumor growth [161]. A key factor for the reactivation of latent CMV is the inflammatory environment. It has been suggested that the inflammatory cytokine IL-6 increases IE expression, cell transition into late-stage gene expression, and the infectivity of the virions produced from latently infected cells [162,163]. Under the influence of IL-3, IL-4, IL-6, M-CSF and G-CSF, infected monocytes harboring latent CMV differentiate into macrophages and dendritic cells and simultaneously reactivate CMV production [163]. IL-6 expression is increased through NF-κB pathway activated by CMV US28 feedback loop. NF-κB-mediated activation of STAT3 and subsequent IL-6 and VEGF expression establish a proliferative signaling, primarily governed by US28 [164]. There is a possibility that the presence of CMV in OC specimens may be a result of the inflammation-induced reactivation of the virus. 

EBV is a herpesvirus which infects over 90% of the world’s population before adolescence and which has been associated with human malignancies of epithelial, hematolymphoid, and mesenchymal derivation [165]. It has been associated with nasopharyngeal carcinoma, Burkitt’s lymphoma, and Hodgkin’s lymphoma. EBV appears to play a direct oncogenic role through genome-wide alteration of promoter methylation and expression of miRNA and genes involved in cell motility and transformation pathways [165,166]. EBV DNA has been detected in approximately 5%–7% of EOC tissue samples [74,76], while no DNA virus transcripts were detected in ovarian serous cystadenocarcinoma [75]. Moreover, no association between EOC and EBV antibody levels has been found [167]. It has been suggested that primary EBV infection at a later age may play a role in the etiology of ovarian cancer. An EBV viral miRNA, miR-BART7, induces cisplatin resistance and develops resistance to first-line chemotherapy and early death in patients with serous OC [76]. EBV nuclear antigens (EBNAs) and membrane proteins (LMP1, LMP2A, and LMP2B) are known target the p53 family in lymphomas and nasopharyngeal and gastric cancer [168]. However, the association between EBV and p53 isoforms in OC is yet to be defined. 

A low prevalence does not exclude the viral initiation or promotion of tumorigenesis, as described by the “hit and run” hypothesis. It proposes that viruses initiating disease can be lost entirely, obscuring a cancer’s viral origin. The “epigenetic signature” left by a tumor virus on the host cell epigenome may help to identify viral “hit and run” oncogenic events, even after the complete loss of tumor virus genomes from neoplastic cells [86]. The virus-specific epigenetic signatures are the molecular memory which persists after the loss of the viral genome in neoplastic cells. During the “hit” phase, the expression of the EBV oncoprotein LMP1 upregulates the activation of epigenetic markers such as cellular DNA methyltransferases (DNMTs) 1, 3a, and 3b, leading to the hypermethylation of E-cadherin [86]. The upregulation of DNMTs and neoplastic transformation under such epigenetic dysregulation may contribute to the EBV-mediated “hit and run” hypothesis. The study of the epigenetic markers and the copy number of the viral genome in the ovarian tumor and non-cancerous tissues’ surroundings may shed some light on the potential role of viruses in OC.

## 6. Conclusions

The ovary and its neighboring parts in the female genitalia are highly susceptible to pathogenic infection. We aim to shed light on viral infections that were detected in ovarian tissues and may hold potential in the process of carcinogenesis. However, the role of viruses in the initiation, promotion, and progression of OC is unknown. The oncomodulatory and tumor-promoting inflammatory mechanisms that these viruses instigate may largely promote the development of a long-term, asymptomatic cancer. The detection of viral sequences does not prove pathogenicity as viral sequences are commonly detected in non-malignant tissues. There is a possibility that the TME provides a favorable milieu for the survival of viruses. In addition, viruses interact with the host’s innate immunity, immune sensors, and tumor signaling that control both viral infection and cancer. The identification of markers that favor the survival of the virus and the downstream molecules affected by the virus may help to elucidate the pathogenicity of the virus. The exploration of the causation of the virus–host interactions may lead to additional strategies for prevention and treatment.

If the HPV and/or herpesviruses are drivers of OC pathogenesis, the natural question appears: what therapeutic strategies could be used to manage OC patients? So far, vaccination with DCs pulsed with modified or unchanged tumor-associated antigens (TAAs) has been tested in plenty of immunotherapy trials devoted to advanced OC; however, this has had hardly satisfactory results [169,170]. Another approach to the treatment of patients with OC is based on the vaccination with both long and short peptides (e.g., p53, Her-2/Neu, and NY-ESO-1 or survivin, WT1, and Ca125, respectively) or, alternatively, with carbohydrate epitopes (e.g., GM2, Globo-H, Levis y, sialyl-Tn, and Thompson Friedreich antigen) conjugated to the carrier protein KLH [171]. In many trials, vaccination with recombined viruses has been tested; however, the use of viruses was limited to the role of vectors for TAAs, such as CEA or MUC1. Some initial preclinical trials performed on mice revealed that empty viral capsids of Cowpea mosaic virus (eCPMV VLPs) used for inhalation reduced the formation of metastatic melanoma tumors in lungs, as well as inhibiting metastatic breast, colon, and ovarian cancer [172]. Some researchers have recommended HPV vaccination to reduce the risk of OC [67], but clinical trials using HPV for vaccination against OC are lacking. The knowledge that a subpopulation of OC expresses non-human genes originating from viruses could be used for designing targeted therapies. They could be designed as either immunotherapies, augmented with viral antigens to treat advanced OC, or as vaccinations, used for the prevention of a subpopulation of OC. CMV-specific immunotherapy (pp65 DC vaccination) and anti-CMV treatment with valganciclovir have shown promise for glioblastoma patients, and these strategies are currently being evaluated in clinical randomized trials [173,174]. The assessment of the efficacy of these vaccines and immune checkpoint inhibitors for OC patients may beget promising preventative measures against OC [175].

## Figures and Tables

**Table 1:** Virus detection in ovarian cancer.

Histology of Tumors ^a^	Prevalence; n (%)	Virus	Detection Method	Reference	Year
Epithelial ovarian adenocarcinoma, Epithelial ovarian tumor of low malignant potential, Epithelial ovarian adenoma	0/18 (0)	HPV6, 16, 18, 31, 35	Southern hybridization	Leake, J. F.; et al. [57]	1989
		HPV6, 11	PCR		
Serous OC	1/7 (14.28)	HPV16, 18	PCR (*E6* gene), Southern hybridization	Lai, C. H.; et al. [58]	1992
Mucinous OC	1/3 (33.3)				
Mixed (serous+mucinous OC)	1/1 (100)	HPV18			
Serous, Endometrioid, Mixed Undifferentiated, Clear cell, Malignant mixed Mullerian tumor with heterologous elements	0/28	HPV16, 18	PCR (*L1* and *E6* genes), dot blot	Runnebaum, I. B.; et al. [59]	1995
Serous, Mucinous, Endometrioid, Clear cell, Brenner, Mixed epithelial, Unclassified epithelial	0/98	LR-HPV types (6, 11, 42, 44, 51, 53, 54, 55, 67, 68, 70) and HR-HPV types (16, 18, 31, 33, 35, 39, 45, 51, 52, 54, 56, 58, 66)	PCR (*L1* gene), ISH	Anttila, M.; et al. [60]	1999
EOC	26/50 (52) 18/50 (36)	HPV16	ISH (*E6* gene) IHC (E6)	Wu, Q. J.; et al. [61]	2003
Malignant ovarian tumor	15/40 (37.5)	Not specified	ISH, IHC	Kuscu, E.; et al. [62]	2005
Benign ovarian tumor	9/32 (28.1)				
Serous cystadenocarcinoma	8/76 (10.5)	HPV16, 33	PCR (*L1* gene); DNA sequencing	Atalay, F.; et al. [63]	2007
Epithelial ovarian neoplasms	3/71 (4.2)	Not specified	PCR (*L1* gene)	Giordano, G.; et al. [64]	2008
Serous adenocarcinoma	3/12 (25)	HPV6	Nested PCR (*E6* and *E7* genes)	Shanmughapriya, S.; et al. [65]	2012
Mucinous adenocarcinoma	0/6 (0)				
Endometrioid adenocarcinoma	3/6 (50)				
Borderline serous OC	6/6 (100)				
Serous OC	2/35 (5.7)	HPV16	PCR (*L1* gene); DNA sequencing	Malisic, E.; et al. [66]	2012
Mucinous OC	1/2 (50)				
Endometrioid carcinoma	1/7 (14.28)				
Ovarian carcinoma	42/100 (42)	HPV16, 18, 45	Nested PCR (*L1* gene; DNA sequencing	Al-Shabanah, O. A.; et al. [67]	2013
Non-cancerous tissue surrounding tumor	8/100 (8)	HPV6, 11			
EOC	2/20 (10)	HPV16	PCR (*E6* gene)	Bilyk, O. O.; et al. [68]	2014
	2/20 (10)	HPV18			
	4/20 (20)	HPV16/18			
Serous, Endometrioid, Mucinous, Clear-cell	1/191 (0.52)	HPV18	qRT-PCR (*E6/E7* genes)	Ingerslev, K.; et al. [69]	2016
EOC	5/100 (5) 4/100 (4) 1/100 (1)	HPV16 HPV18 HPV33	Nested PCR (*L1* gene); DNA sequencing	Hassan, Z. K.; et al. [70]	2017
EOC	40/99 (40.4)	HPV2, 4, 5, 6b, 7, 10, 16, 18, 32, 48, 49, 50, 60, 54, 92, 96, 101, 128, 129, 131, 132	Microarray-based method, PathoChip Array	Banerjee, S.; et al. [56]	2017
Non-cancerous tissue surrounding tumor	20/20 (100)	HPV41, 88, 53, 103			
EOC	19/27 (70.4) 2/27 (7.4) 1/27 (3.7)	HPV16 HPV6 HPV45	Nested PCR (*E6* gene); DNA sequencing	Paradowska, E.; et al. [71]	2019
Metastatic ovarian tumors	4/ 4 (100)	HPV16			
Benign ovarian tumors	2/8 (25)				
Serous adenocarcinoma	6/12 (50)	CMV	Nested PCR (*UL55* gene)	Shanmughapriya, S.; et al. [65]	2012
Mucinous adenocarcinoma	3/6 (50)				
Endometrioid adenocarcinoma	3/6 (50)				
Borderline serous OC	3/6 (50)				
Serous adenocarcinoma and other histotypes	34/45 (75.5)	CMV (IE)	IHC (IE and pp65)	Rådestad et al. [72]	2018
	11/42 (26.2)	CMV (pp65)			
Benign ovarian cystadenoma	20/30 (67)	CMV (IE)			
	4/29 (14)	CMV (pp65)			
HGSOC with prechemotherapy	8/10 (80)	CMV (IE)	IHC (IE and pp65), ISH (DNA ß2.7)	Carlson et al. [73]	2018
	4/10 (40)	CMV (pp65)			
	3/3 (100)	CMV (DNA)			
HGSOC with neoadjuvant chemotherapy	4/9 (44)	CMV (IE)			
	5/8 (62.5)	CMV (pp65)			
	5/5 (100)	CMV (DNA)			
EOC Metastatic ovarian tumors Benign ovarian tumors	19/27 (70.4) 4/4 (100) 0/8 (0)	CMV	Nested-PCR (*US28* gene), DNA sequencing	Paradowska, E.; et al. [71]	2019
Serous, mucinous, and endometrioid adenocarcinomas, clear–cell carcinomas	1/191 (0.5)	CMV	qRT-PCR, DNA sequencing	Ingerslev, K.; et al [74]	2019
Ovarian serous cystadenocarcinoma	0/419 (0)	EBV	RNA-Seq	Khoury, J.; et al. [75]	2013
Serous OC	38/487 (7.8)	EBV	miRNA expression, qRT-PCR	Pandya, D.; et al. [76]	2014
EOC	0/27 (0)	EBV	qRT-PCR, nested PCR	Paradowska, E.; et al. [71]	2019
Serous, mucinous, and endometrioid adenocarcinomas, clear–cell carcinomas	10/191 (5.2)	EBV	qRT-PCR, DNA sequencing, ISH	Ingerslev, K.; et al [74]	2019

^a^ Histology of tumors as originally described by cited authors; n, number of cases with the virus genotype; HPV, human papillomavirus; CMV, cytomegalovirus; EBV, Epstein-Barr virus; PCR, polymerase chain reaction; qRT-PCR, quantitative-real-time PCR; ISH, In situ hybridization; IHC, immunohistochemistry; OC, ovarian cancer; EOC, epithelial ovarian cancer; HGSOC, high grade serous ovarian carcinoma.
